# The Social Brain and Emotional Contagion: COVID-19 Effects

**DOI:** 10.3390/medicina56120640

**Published:** 2020-11-25

**Authors:** Anna Valenzano, Alessia Scarinci, Vincenzo Monda, Francesco Sessa, Antonietta Messina, Marcellino Monda, Francesco Precenzano, Maria Pina Mollica, Marco Carotenuto, Giovanni Messina, Giuseppe Cibelli

**Affiliations:** 1 Department of Clinical and Experimental Medicine, University of Foggia, 71122 Foggia, Italy; anna.valenzano@unifg.it (A.V.); francesco.sessa@unifg.it (F.S.); giuseppe.cibelli@unifg.it (G.C.); 2 Department of Education Sciences, Psychology, and Communication, University of Bari, 70121 Bari, Italy; alessia.scarinci@uniba.it; 3 Department of Experimental Medicine, Section of Human Physiology and Unit of Dietetics and Sports Medicine, Università degli Studi della Campania “Luigi Vanvitelli”, 80100 Naples, Italy; vincenzo.monda@unicampania.it (V.M.); anto.messina83@gmail.com (A.M.); marcellino.monda@unicampania.it (M.M.); 4 Clinic of Child and Adolescent Neuropsychiatry, Department of Mental Health, Physical and Preventive Medicine, Università degli Studi della Campania “Luigi Vanvitelli”, 80100 Naples, Italy; francesco.precenzano@unicampania.it (F.P.); mariapina.mollica@unina.it (M.C.); 5 Department of Clinical and Experimental Medicine, University of Naples, 80138 Naples, Italy; marco.carotenuto@unicampania.it

**Keywords:** central nervous system, coronavirus, emotional contagion, pandemic, COVID-19

## Abstract

Background and objectives: Coronavirus disease 2019 (COVID-19) is a highly contagious infectious disease, responsible for a global pandemic that began in January 2020. Human/COVID-19 interactions cause different outcomes ranging from minor health consequences to death. Since social interaction is the default mode by which individuals communicate with their surroundings, different modes of contagion can play a role in determining the long-term consequences for mental health and emotional well-being. We examined some basic aspects of human social interaction, emphasizing some particular features of the emotional contagion. Moreover, we analyzed the main report that described brain damage related to the COVID-19 infection. Indeed, the goal of this review is to suggest a possible explanation for the relationships among emotionally impaired people, brain damage, and COVID-19 infection. Results: COVID-19 can cause several significant neurological disorders and the pandemic has been linked to a rise in people reporting mental health problems, such as depression and anxiety. Neurocognitive symptoms associated with COVID-19 include delirium, both acute and chronic attention and memory impairment related to hippocampal and cortical damage, as well as learning deficits in both adults and children. Conclusions: Although our knowledge on the biology and long-term clinical outcomes of the COVID-19 infection is largely limited, approaching the pandemic based on lessons learnt from previous outbreaks of infectious diseases and the biology of other coronaviruses will provide a suitable pathway for developing public mental health strategies, which could be positively translated into therapeutic approaches, attempting to improve stress coping responses, thus contributing to alleviate the burden driven by the pandemic.

## 1. Introduction

A pandemic has to be considered in terms of a global social phenomenon. To understand the pathophysiology involved and recommend actions to prevent and contain the spread of the disease, media and public health primarily concentrated on pathogens and biological threats [1]. However, mental health concerns assumed a secondary relevance, although it is not yet clear whether the etiology of the neurological and psychiatric symptoms observed in patients with COVID-19 [2] was attributable to the virus itself, to the stress related to a pandemic, or to pharmacological treatment [3]. Several countries have enacted different countermeasures in order to contain the infection and prevent health systems from becoming overwhelmed. The most important action was the so-called “lockdown”; this restriction has been applied in 82 countries, generating severe socioeconomic consequences. Moreover, the lockdown combined with the fear of COVID-19 has caused physical, emotional, and psychological distress [4]. Indeed, social isolation, loneliness, changes in daily habits, and financial insecurity—direct consequences of social distancing measures—were all risk factors linked to the onset of major depressive and post-traumatic stress disorder, with potentially long-lasting effects on brain functions. Thus, understanding the mental implication of a pandemic becomes crucial for the effective management of the current pandemic, through the awareness that feelings such as fear, anxiety, and anger represent its essential components [5]. Another issue that deserves immediate attention concerns the sense of fear generated by the easy access to various social media sites, which facilitates the dissemination of information about virus transmission, number of people with COVID-19, and mortality rate, thus worsening a dysphoric mental condition [6,7]. Therefore, while medical and public health professionals strive to contain the spread of the pandemic, people must manage a different kind of contagion, the emotional contagion, a mechanism by which people’s emotions—positive or negative—become “viral” within groups, influencing thoughts and actions. The psychological distress is closely related to viral epidemics: for example, during the Spanish flu pandemic, several psychiatric complications were reported [8]. Moreover, several recent studies performed during the COVID-19 outbreak reported various psychiatric symptoms such as extreme fear, a growing degree of uncertainty, problems of loneliness, limitation of degree of freedom that could lead to a dramatic mental health burden [9,10].

In this brief review, we focused on some basic aspects of human social interaction, emphasizing some particular features of the emotional contagion. Moreover, we analyzed the main reports that described brain damage related to the COVID-19 infection.

## 2. Interplay between Infectious and Emotional Contagion

The term contagion refers to the transmission of an infectious disease from a sick person to a healthy one, either directly or through polluted vehicles such as air, water, food, excretions, or living carriers of pathogenic microorganisms. In the psychological field, contagion represents the transmission of ideas, beliefs, feelings, or moods from one individual to another and, at the psychiatric level, the contagion is considered as the pathological equivalent of suggestion; while, at the social level, the contagion is taken into consideration for its value of determining particular modifications in a social context. According to the appraisal theory of emotion, emotions are responses that reflect a person’s assessment of how significant something in the environment is for their well-being [11]. As fear and anxiety, characterizing the COVID-19 pandemic, are predominantly negative emotions, the spread via social media of such a negative feeling could lead to an emotional contagion, fostering a negative emotional climate, which is further amplified by the social media rewards of emotionally charged messages. People are usually deeply concerned about job security and personal freedom, thus perceiving the pandemic as a threat to these things. Another important feature of emotions is that they have particular action-tendencies [12]. Thus, fear for their life, health, and livelihood will likely motivate some people to take protective actions. Furthermore, emotional contagion has consequences that extend beyond how people feel, because emotions influence how people think and act [11]. Perception of emotion seems to include neural mechanisms that would generate similar emotions in observers [13,14,15,16], allowing them to share the emotional state of another individual. Emotional contagion might be considered a precursor of empathy, which provides information on the mental state of other people, as well as the motivation for cooperative behavior and communication [17]. The emotional contagion can, therefore, represent a first step in the mentalization process, which is understood as the ability to consider the behavior of others as the result of a mental state similar to one’s own and as the ability to recognize one’s own existence and to regulate one’s behavior based on another.

Although several studies have proved that the COVID-19 outbreak might have generated different forms of psychological distress from mild to moderate-to-severe symptoms, it is not clear why some people are more affected than others. A possible explanation is that while anxious individuals declare excessive discomfort to ensure care, individuals with an avoidable attachment may appear to be very calm in an uncomfortable situation, while their internal experience may be the opposite. Alternatively, individuals with marked characteristics of avoidable attachment, who tend to be self-directed and often show no anxiety following social separation, may perceive self-isolation, as well as preventive social distancing measures, as less stressful than anxiously attached individuals [18]. In light of these considerations, it is important to program “personalized” psychological and mental health support in all countries involved in the COVID-19 outbreak.

## 3. COVID-19 and Brain Damage

In the first period of the COVID-19 pandemic, the lack of postmortem investigations did not allow a prompt definition of the pathways of this infection [19]. Indeed, in its first phase, many governments did not provide uniform tools to perform a sufficient number of autopsies, generating the so-called “lockdown of science” [20,21,22]. Moreover, although in the second phase many autopsies have been performed around the world showing different important evidences about the pathway of this infection, scarce evidence has been collected on brain tissue involvement [23,24,25,26]. Even if healthcare organizations and researchers published guidelines and recommendations about postmortem investigation, frequently minimally invasive autopsies were performed avoiding brain extraction [27]. Postmortem investigations of the central nervous system (CNS) were restricted to a few individual reports and comprised cerebral swelling and focal hemorrhagic injuries scattered throughout the white matter with swollen axons at the borders of the hemorrhagic foci, responsive gliosis, and oligondendrocytic apoptosis around the injuries [28]. Brain pathological characteristics also include microthrombi and acute stroke, a central parenchymal infiltrate of CD3-positive T lymphocytes [29]. Viral molecules in the frontal lobe sections were found under the electron microscope separately or in small vesicles of endothelial cells. The neural-cell bodies also showed cytoplasmic vacuoles that contained enveloped viral components [30]. In a recent postmortem case series, Matschke et al. [31] reported that the neuropathological changes in patients with COVID-19 seemed to be mild and pronounced neuroinflammatory changes in the brainstem were the most common finding. No evidence for CNS damage directly caused by COVID-19 was described. 

Analyzing the CNS clinical symptoms, the patients affected by the COVID-19 infection suffered from the common flu symptoms, such as headache, myalgia, impaired consciousness, and encephalopathy. Nevertheless, the almost exclusive symptoms were anosmia and ageusia [32]. In severely ill COVID-19 patients, several neuropathological features were described, such as vascular and demyelinating etiologies, with specific manifestations (cerebral edema, hemorrhagic white matter lesions, peripheral axonal injury, and demyelination damage with macrophage infiltration) [30,33]. Furthermore, the broad spectrum of symptoms could include, in severe cases, neurogenic respiratory failure, encephalopathy, silent hypoxemia, generalized myoclonus, neuroleptic malignant syndrome, and Kawasaki syndrome [34,35].

## 4. Brain Changes and Neural Substrates of the Social Brain

Much evidence has documented that COVID-19 can cross the blood–brain barrier or reach the brain via the olfactory bulb. Although it has been shown that the virus can interact with ACE2 (Angiotensin-converting enzyme 2) receptors [36], due to the virus spike protein S1, to attach to the host membrane of neurons, endothelial cells, kidneys, lungs, and small intestine [37], COVID-19 seems to be able to penetrate the brain also by hematogenous spread, directly through the cribriform plate or through retrograde neuronal synapses from the olfactory bulb and vagal afferents [38,39]. On the other hand, the interaction with ACE2 cerebral receptors may be involved in epileptogenesis also sustained by pro-inflammatory cytokine dismission that causes blood–brain barrier disruption, increase of glutamate and aspartate and reduction of GABA levels, with a consequent impairment of ion channel function [40]. Moreover, brain changes linked to COVID-19 seem to be multiple and may include inflammation and cerebrovascular events, headache and dizziness, encephalopathy, meningoencephalitis, acute hemorrhagic necrotizing encephalopathy, and intracerebral hemorrhage [41,42]. In general, the cytokinin storm may affect cerebral areas independently of age and causative events with impairment of connectomic organization also in the limbic system [43].

**Figure 1:**
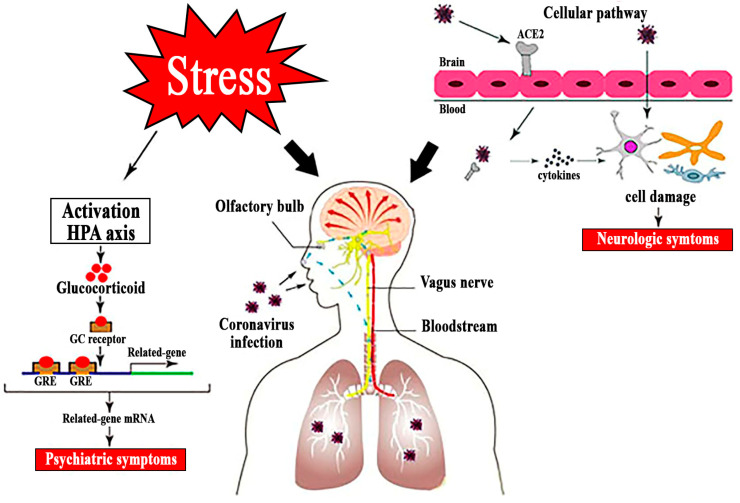
Brain changes linked to the coronavirus disease 2019 (COVID-19) infection.

To date, various biological alterations associated with the coronavirus infection have been identified, and some of them, especially those related to microglia activation [44,45] and cytokine signaling [46,47], might be of relevance to specific mental health outcomes (as shown in Figure 1). As well as having extensive connections to other brain regions, including the amygdala, orbitofrontal cortex, and hippocampus, which play a major role in emotion, learning and memory, the olfactory bulb is rich in dopamine, which is important for pleasure, motivation, and action.

A restricted set of brain regions has been shown in monkeys and confirmed by brain imaging in humans [48,49] that is related to social cognition. This set of regions was named the “social brain”. To ask the question what is the social brain for, we might consider that the function of the social brain is to enable us to make predictions during social interactions, not necessarily being conscious and deliberated [50]. Our social brain has two problems to solve. First, it must read the mental state of the person we are interacting with. Second, it must make predictions about future behavior on the basis of that mental state. However, whatever the mechanism, the result is that behaviors are contagious. 

**Figure 2:**
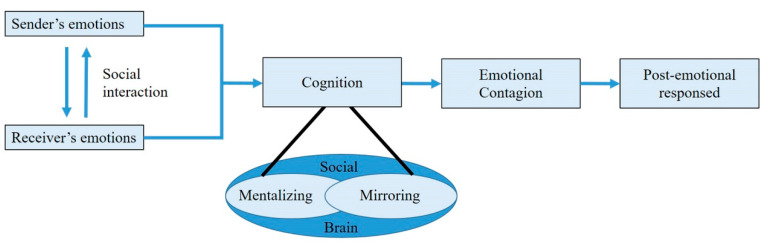
Neural pathway involved in emotional processing.

As its major components, the social brain includes the amygdala, orbital frontal cortex, and temporal cortex. However, two more regions must be considered: the medial prefrontal cortex (MPFC) and the adjacent paracingulate cortex [51] and a “mirror” system, which allows us to share the experiences of others [52]. Mirroring and mentalizing are two basic mechanisms that have been proposed to support the function of mind “reading”. These mechanisms work together in a complementary way but with different temporal scales [53]. Mirroring is fast, implicit, automatic, and intuitive, and it is considered to contribute at the subconscious level to the understanding of other people’s intentions or goals [54]. In contrast, mentalizing is explicit, controlled, conscious, reflective, and, therefore, slower than mirroring [55]. Because the mirroring and mentalizing brain circuits only overlap partially [56], they can operate in parallel and even independently, although they usually participate in the same tasks. A schematic representation of the emotional contagion via social interactions is shown in Figure 2.

The main circuitry of motor mirroring, also called the action–perception network, comprises the premotor areas in the inferior frontal cortex and a compact frontoparietal network combining visual, somatosensory, and motor processing; the system has also close connections to limbic areas [52]. The network of brain regions supporting mentalizing includes the temporoparietal junction, which has been associated with inferring temporary states of others [57]; the MPFC, which comprises functional subregions involved either in more cognitive or emotional tasks [51,58]; and the superior temporal sulcus, which is especially sensitive to movement [59] and responds to a wide range of social stimuli [60]. All these circuitries work together with other brain areas and networks that support social cognition. Moreover, several brain areas including sensorimotor cortices support shared sensorimotor representations for self and non-self. The striatum is also important as it is related to reward learning, as well as the prefrontal cortex that controls meta-cognition and higher-order thinking [49].

## 5. Discussion and Conclusions

COVID-19 can cause several significant neurological disorders, and the pandemic has been linked to a rise in people reporting mental health problems, such as depression and anxiety. In this review, we summarized the available data of a set of brain regions, namely, the social brain, that are dedicated to social cognition, emphasizing its putative role in promoting emotional contagion, as a consequence of the COVID-19 pandemic. Moreover, we have reviewed the data about brain damage related to the COVID-19 infection, detected during postmortem investigations.

More recently, neurological and neurocognitive features of the COVID-19 infection have become a major concern. Neurocognitive symptoms associated with COVID-19 include delirium, both acute and chronic attention and memory impairment related to hippocampal and cortical damage, as well as learning deficits in both adults and children [2,61]. The percentage of COVID-19 patients with neurological disorders varies greatly between studies. One of the explanations is the inconsistent method used for the evaluation. Research has shown that behavioral evaluation (e.g., patient’s visual responses, motor function, and communication) is often inaccurate [62]. In this way, objective assessments of brain function can help to pinpoint when neurocognitive symptoms begin to appear in COVID-19 patients. Further studies should be performed to clarify these important aspects in COVID-19 survivors. Moreover, the adverse effects on the brain systems of the survivor, linked to the use of pharmaceutical drugs or ventilators, should be investigated. Particularly, scientists and clinicians should better understand the type of damage it leaves behind. 

As highlighted in this paper, the COVID-19 infection may be related to different types of brain damage. Several studies reported the postmortem findings on the brain tissue of subjects who had died from/with the COVID-19 infection, describing responsive gliosis and oligondendrocytic apoptosis in different areas. It is well known that different brain areas control several important functions such as breathing, moving, resting, and feeding and create our experiences of emotion. Brain areas in the frontal lobe have been implicated in behavioral flexibility and control: past work found target recognition signals in areas of the ventral visual cortex and in subregions of the parietal and frontal cortex [63,64,65]. In this scenario, a direct link may be suggested among COVID-19 infection, brain damage, and behavioral impairment.

The pandemic has been especially considered in terms of a global social phenomena, during which public mental health can be affected because of multiple reasons, since the level of negative emotions and sensitivity to social risks increases; while the scores of positive emotions and the level of life satisfaction decrease. Because of the fundamental importance of interpersonal interaction on human behavioral patterns, we argue that brain mechanisms related to “social” emotions should be reconsidered during natural social interaction, contributing to improve our understanding of the neuronal underpinning of related mental disorders. The worrying number of COVID-19 cases (over 38 million cases worldwide, as of 13 October 2020; https://www.worldometers.info/coronavirus) raised the question as to whether COVID-19 uses a more sophisticated strategy to ensure its rapid spread all over the world, by modulating human behavior. In this respect, special emphasis must be placed on host/parasite interactions, which constitute a well-known dynamic concept, leading to abnormal behavioral patterns in the host, which are advantageous either to the host, such as employing defensive behavior, or to the parasite, as a manipulative behavior [66]. Understanding any such strategy is fundamental to keep the viral spread under strict control. Although our knowledge on the biology and long-term clinical outcomes of the COVID-19 infection is largely limited, approaching the pandemic, based on lessons learnt from previous outbreaks of infectious diseases and the biology of other coronaviruses, will provide a suitable pathway for developing public mental health strategies, which could be positively translated into therapeutic approaches, attempting to improve stress coping responses, thus contributing to alleviate the burden driven by the pandemic. Therefore, considering the effects of the COVID-19 infection on the mental health and well-being of the general population, health professionals, and vulnerable people, all governments should provide helpful interventions of mental health professionals [67,68]. Particularly, their involvement would be very important in the management of this dangerous situation, preventing the development of widespread, full-blown psychiatric disorders, which would be an additional social and economic burden on the looming post-epidemic crisis. 

Finally, considering that healthcare workers are a category particularly exposed to the distressing situation related to COVID-19 outbreaks, it is important to have several initiatives to support people’s mental health. A health problem for these people must be avoided both for their health status and for the health system that could be further placed under pressure because of the absence of health professionals [69,70]. For example, in China, several initiatives, such as leisure activities and training on how to relax, were properly arranged to help staff reduce stress. Psychological counselors were activated to listen to difficulties or stories encountered by staff at work, providing adequate support [71].
